# Seasonality and weather influence on mixed episodes of bipolar disorder

**DOI:** 10.1192/j.eurpsy.2025.309

**Published:** 2025-08-26

**Authors:** F. Cunha, E. Almeida, N. Castro, J. Abreu, R. Cabral, D. Teixeira

**Affiliations:** 1 ULS Viseu Dão-Lafões, Viseu; 2 ULS Cova da Beira, Covilhã, Portugal

## Abstract

**Introduction:**

Seasonal patterns in bipolar disorder episodes, particularly manic episodes, have been widely studied, revealing a peak in early spring and a decline in late fall. However, less attention has been given to the seasonal variation of mixed states. Mixed episodes, a subtype of bipolar disorder, are characterized by both manic and depressive symptoms. Recent studies have suggested that mixed episodes may follow a different seasonal pattern compared to pure manic episodes, peaking in late summer. Understanding this seasonality can offer valuable insights into the timing and management of hospital admissions for bipolar disorder. In this study, we conducted a retrospective analysis of hospital admissions for bipolar disorder, evaluating the influence of meteorological factors on the frequency of admissions, particularly for mixed episodes.

**Objectives:**

This study aims to analyze the prevalence of hospital admissions due to bipolar disorder at the Psychiatry Service of ULS-VDL during 2021 and 2022, with a particular focus on mixed episodes. Additionally, it seeks to explore the relationship between meteorological factors, such as temperature and cloud cover, and the frequency of admissions, identifying any seasonal patterns associated with bipolar episodes. This analysis aims to explore the potential link between environmental changes, such as temperature and cloud cover, and the occurrence of mixed states in bipolar disorder.

**Methods:**

A retrospective analysis was conducted of hospital admissions for bipolar disorder between 2021 and 2022 (n=71) at the Psychiatry Service of ULS-VDL. Monthly averages of meteorological data (temperature, precipitation, cloud cover, and solar radiation) were obtained from a local weather station. Poisson regression was used to assess the relationship between monthly admissions and meteorological variables.

**Results:**

Of the 71 total episodes, 16 were mixed episodes of bipolar disorder. The results showed a statistically significant association between decreased temperature (IRR=0.843, 95% CI [1.568-0.118], p=0.023) and increased cloud cover (IRR=0.162, 95% CI [0.023-0.302], p=0.023), compared to the previous month, and an increase in admissions for mixed episodes.

**Conclusions:**

The findings of this study indicate a significant association between decreases in temperature and increases in cloud cover with higher rates of hospital admissions for mixed episodes of bipolar disorder. These results highlight the potential influence of meteorological factors on the seasonality of mixed episodes. Further research is needed to confirm these patterns and to explore their implications for the management and prevention of bipolar disorder hospitalizations.

Note: We intend to increase the number of years included and episodes.

**Disclosure of Interest:**

None Declared

